# GUIdock: Using Docker Containers with a Common Graphics User Interface to Address the Reproducibility of Research

**DOI:** 10.1371/journal.pone.0152686

**Published:** 2016-04-05

**Authors:** Ling-Hong Hung, Daniel Kristiyanto, Sung Bong Lee, Ka Yee Yeung

**Affiliations:** Institute of Technology, University of Washington, Tacoma, WA 98402, United States of America; UGent / VIB, BELGIUM

## Abstract

Reproducibility is vital in science. For complex computational methods, it is often necessary, not just to recreate the code, but also the software and hardware environment to reproduce results. Virtual machines, and container software such as Docker, make it possible to reproduce the exact environment regardless of the underlying hardware and operating system. However, workflows that use Graphical User Interfaces (GUIs) remain difficult to replicate on different host systems as there is no high level graphical software layer common to all platforms. GUIdock allows for the facile distribution of a systems biology application along with its graphics environment. Complex graphics based workflows, ubiquitous in systems biology, can now be easily exported and reproduced on many different platforms. GUIdock uses Docker, an open source project that provides a container with only the absolutely necessary software dependencies and configures a common X Windows (X11) graphic interface on Linux, Macintosh and Windows platforms. As proof of concept, we present a Docker package that contains a Bioconductor application written in R and C++ called networkBMA for gene network inference. Our package also includes Cytoscape, a java-based platform with a graphical user interface for visualizing and analyzing gene networks, and the CyNetworkBMA app, a Cytoscape app that allows the use of networkBMA via the user-friendly Cytoscape interface.

## Introduction

Reproducibility is a vital feature in science [1, 2]. Recent articles in the June 26 issue of Science discussed how rarely published results can be reproduced across different disciplines [2, 3]. Nosek and colleagues proposed guidelines consisting of eight standards and three levels to promote transparency, openness and reproducibility in scientific publications [1]. These guidelines progress from level 0 to level 3 and become increasingly stringent for each standard (see “proposed standards and references” in the Supplementary Material of Nosek *et al*. [1] for details). Computational method development and data analyses have become integral to many disciplines, such as biomedical research. For data analyses and software implementations, level 2 of the Nosek *et al*. guidelines requires that the code must be posted to a trusted repository. In level 3, they propose the additional requirement that the reported analyzes be reproduced independently before publication.

These suggestions overlook the fact that modern biomedical workflows and pipelines consist of multiple applications and libraries, each with their own set of software dependencies. Hence, suites such as Bioconductor [4], BioPython [5], and BioPerl [6] where the user is assured that the dependencies for the components are properly installed have become increasingly popular. The obvious drawback to this approach is that one is limited to the components included in the suite. In addition, reproducing workflows that use interactive graphics remain problematic as each operating system uses their own graphical environment. Our solution to this problem is GUIdock, which allows for replication of the application, graphics and software environments that produced the analytic results reported in scientific publications.

GUIdock uses Docker https://www.docker.com/, an open source project that incorporates a light weight Linux wrapper (container) to ensure application portability and infrastructure flexibility. On a Linux host, Docker uses the host system. On Mac OS and Windows systems, a single Docker container consists of a Virtual Machine (VM) containing the guest software and its Linux environment. Containers differ from traditional VMs in that the resources of the operating system (OS) and not the hardware are shared transparently (virtualized). Multiple containers share a single OS kernel saving considerable resources. Docker also supports Dockerfiles that contain the instructions to build a Docker Image from scratch or another Docker Image. Images can be downloaded from repositories using git https://git-scm.com/ or bundled with the Dockerfile to form packages. Docker provides an easy, modular method to build, distribute and replicate complex pipelines and workflows across multiple platforms.

**Fig 1:**
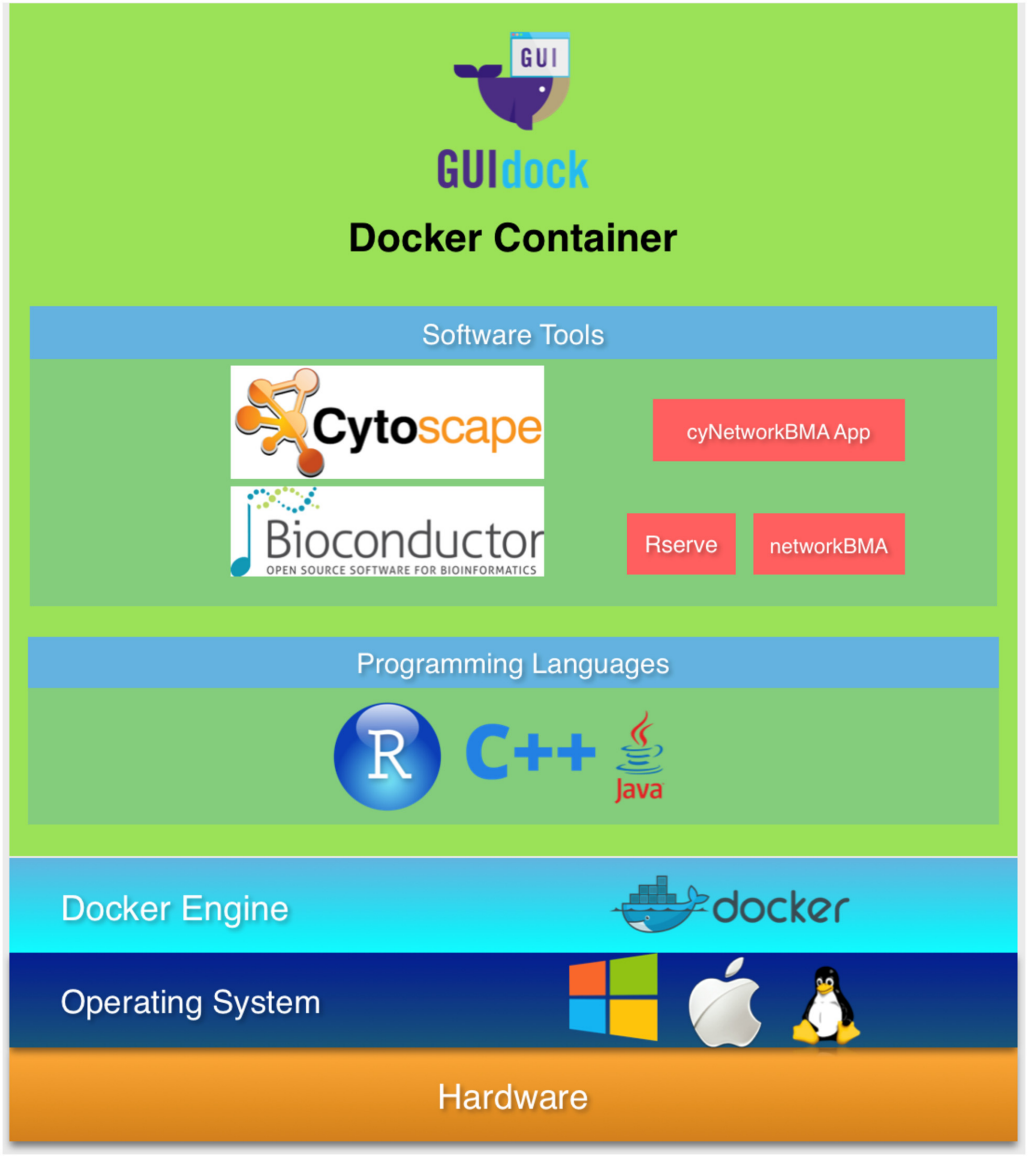
Overview of GUIdock.

Although Docker provides a container with the original computational environment, the host system, where the container software is executed, is responsible for rendering graphics. GUIdock configures an additional X Windows software layer that allows for consistent graphics on a variety of host platforms. Thus a complex bioinformatics pipeline with GUI components originally running on a Linux machine can be replicated and tested on a Windows or Mac OS machine. This greatly facilitates the reproduction of scientific results arising from real-world workflows. Fig 1 shows an overview of GUIdock.

### Related Work

In this work, we showcase GUIdock, a method for deploying containers with a graphical user interface. As proof of concept, our GUIdock package includes our previously published software tools for gene network inference [7–11]. A gene network can be represented by a graph, in which nodes are genes and edges capture relationships between genes. There are many applications for these computationally derived gene networks, such as systems approaches to identify disease genes [12]. Therefore, many computational methods and software tools have been developed to infer gene networks from genome-wide data and subsequently to formulate hypotheses from these computationally derived networks. Excellent review articles have been written to cover these advances in methods and tools, for example, [12–14].

#### Inference of gene networks using Bayesian Model Averaging (BMA)

In regression-based network inference algorithms, we aim to search for candidate regulators (i.e. parent nodes) for each target gene. In other words, we model the target gene’s expression levels as the response variable (*y*), and the candidate regulators’ expression levels as independent variables (*x*′ *s*) in a regression framework. This problem is then reduced to a variable selection problem such that the goal is to identify variables (parent nodes) that can be used to predict the expression levels of the target gene. Many regression-based gene network inference methods have been developed, such as [15–17].

BMA is an ensemble method that accounts for model uncertainty by averaging over the predictions of multiple models [18, 19]. In the context of gene network inference, a model is a set of candidate regulators. We previously showed the effectiveness of using variants of BMA as multivariate variable selection methods in the context of time series gene expression data [7–9].

#### networkBMA

We implemented these BMA network inference methods in R and C++. Our networkBMA package [10] is publicly available from the Bioconductor repository http://bioconductor.org/packages/release/bioc/html/networkBMA.html. The main function networkBMA takes gene expression data as one of the input arguments, allows the specification of prior probabilities to guide the search of optimal parent nodes for each target gene, and outputs an edge list consisting of edges in the form of (parent, child, posterior probabilities) relationships. The user can also specify a posterior probability threshold to filter out edges below the given threshold.

In addition to gene network inference, the networkBMA package also features functions for the assessment of gene networks. Specifically, the contabs.netwBMA function compares the edges in the inferred network to a given set of known regulatory relationships using a contingency table approach, and the scores function computes assessment statistics corresponding to the contingency table, including sensitivity, precision, specificity, recall etc. There are also functions to plot and compute the area under the receiver operating characteristic (ROC) and precision recall (PR) curves.

#### Cytoscape

Visualization and analyses of networks are integral to systems biology research. Cytoscape is a well-established Java-based stand-alone application for analyzing and visualizing networks [20–22]. Cytoscape offers a user-friendly graphical user interface (GUI) for visualizing a given network, and provides an app store at http://apps.cytoscape.org/ from which apps for various systems biology applications can be downloaded [23]. In addition, software developers can submit new apps to be made available from the Cytoscape App Store, such as [11, 24–26]. As an example, cyREST is a RESTful API module for Cytoscape [24], and the Ideker Lab has demonstrated the use of Cytoscape, cyREST and Docker in a meeting focused on visualizing biological data (VIZBI 2015 [27]).

#### CyNetworkBMA app

CyNetworkBMA [11] is available on the Cytoscape App Store at http://apps.cytoscape.org/apps/cynetworkbma. It is an easy-to-use tool that integrates our networkBMA Bioconductor package into Cytoscape, thus allowing the user to directly visualize the resulting gene networks using the Cytoscape utilities. In particular, CyNetworkBMA uses Rserve to integrate with R over a binary protocol on top of TCP/IP [28]. This means Cytoscape and R run in separate processes, potentially on different machines and platforms.

While the CyNetworkBMA app adds an easy-to-use graphical interface and visualization utilities to the BMA-based gene network inference methods implemented in the networkBMA Bioconductor package, there are many steps involved in installing CyNetworkBMA. In addition to Cytoscape and R, CyNetworkBMA depends on multiple R and Bioconductor packages, including networkBMA for network inference and assessment, igraph [29] for algorithms used in removing potential cycles from networks, and Rserve for exposing R services over TCP/IP.

#### Docker for bioinformatics applications

Docker is an emerging platform that is gaining traction in the scientific community [30]. In particular, Rocker is a project containing pre-built Docker images and Dockerfiles to run R using Docker containers [31], and the Bioconductor project has deposited Docker Images in Docker Hub and source Dockerfiles in GitHub [32]. As another example, the BioDocker and BioBoxes project has a GitHub repositories for pre-configured containers with bioinformatics tools [33]. As of September 2015, there are about 15 containers in the BioDocker repository, and most of these containers are built for proteomics and mass spectrometry data analyses. In addition, the Genouest group in France has begun hosting a repository for Docker containers called Bioshadock [34]. Galaxy has an easy-to-use interface and provides a browser-based infrastructure for workflow management. Users can build a workflow in Galaxy and export the workflow to Docker [35, 36].

### Our Contributions

Docker containers have largely been used for non-graphical applications. Containers have *not* been used to distribute the normal GUI based workflows that users are accustomed to. This is due to the difference in high level APIs used for the different windowing environments by the major operating systems. GUIdock addresses this deficiency by configuring a common X11 windowing interface on Linux, Mac OS and Windows. A GUI workflow using X Windows can now be duplicated on most platforms.

We demonstrate the feasibility of using Docker for applications with a GUI, and hence containers that support software tools and data analytic pipelines with a graphical user interface. In particular, we illustrate the use of containers for systems biology applications, including Bioconductor packages written in R and C++, and Cytoscape, a stand-alone java-based application with a graphical user interface. The Docker package we present is a proof-of-concept example that containers can enhance the reproducibility of analytic results produced using applications with graphical interfaces. Our Docker image and Dockerfile are publicly available at https://github.com/WebDataScience/GUIdock.

**Fig 2:**
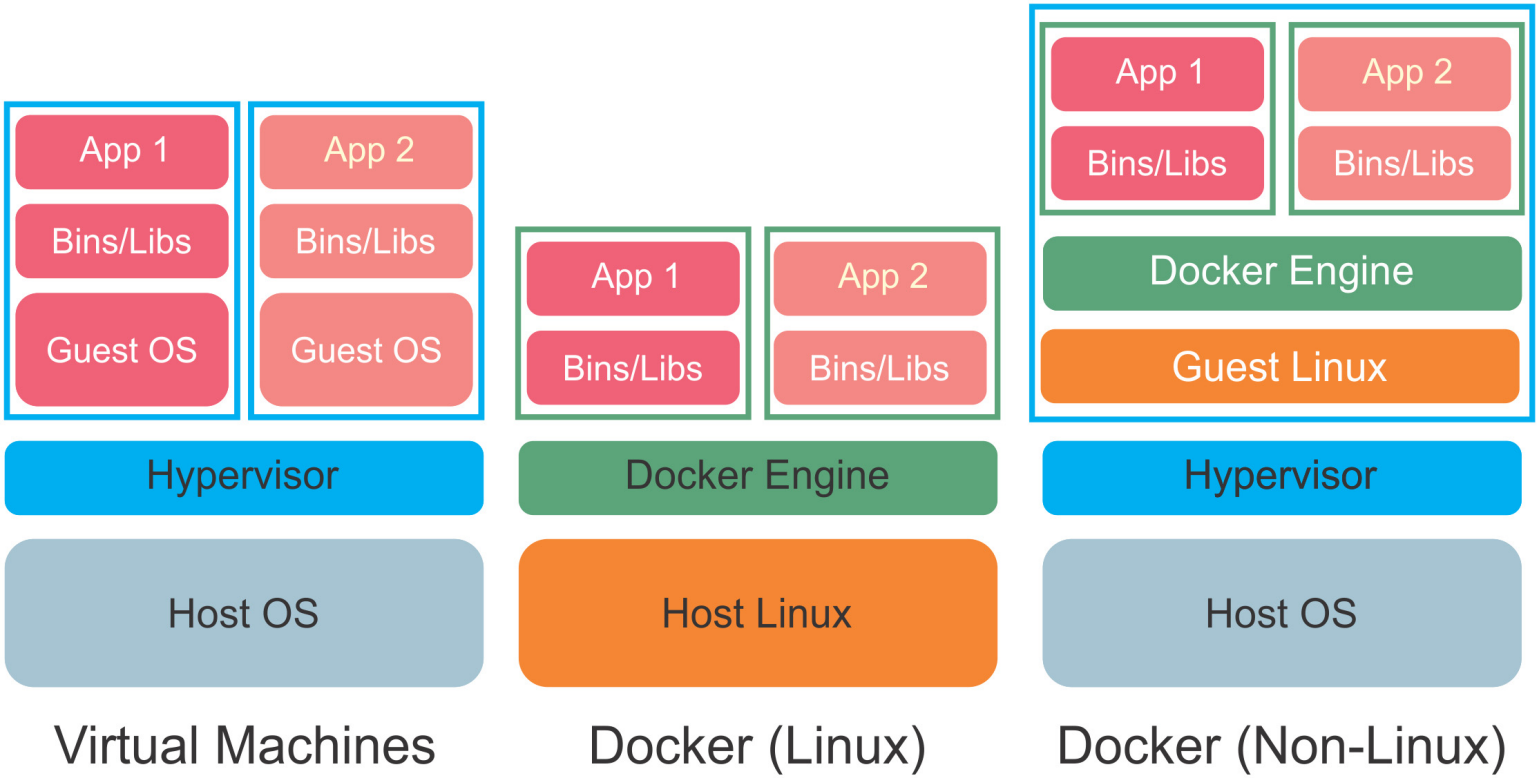
A comparison of the architecture of virtual machines and Docker software containers. Virtual machines are denoted by cyan boxes and software containers are denoted by green boxes. The left stack is a Type-2 virtual machine (VM) which uses a hypervisor to emulate the guest OS. The application software, dependences, and the guest OS are all contained inside the VM. A separate VM, dependencies and guest OS are required for each application stack that is to be deployed. The middle stack depicts Docker container software on a Linux host. Docker uses the host Linux system and packages the application and dependencies into modular containers. No VM is necessary and the OS resources for the two application stacks are shared between different containers. The right stack depicts Docker on a non-Linux system. Because Docker requires Linux, a lightweight VM with a mini-Linux Guest OS is necessary to run Docker and encapsulate the software containers. This still has the advantage that only a single VM and Guest Linux system is required regardless of the number of containers.

**Fig 3:**
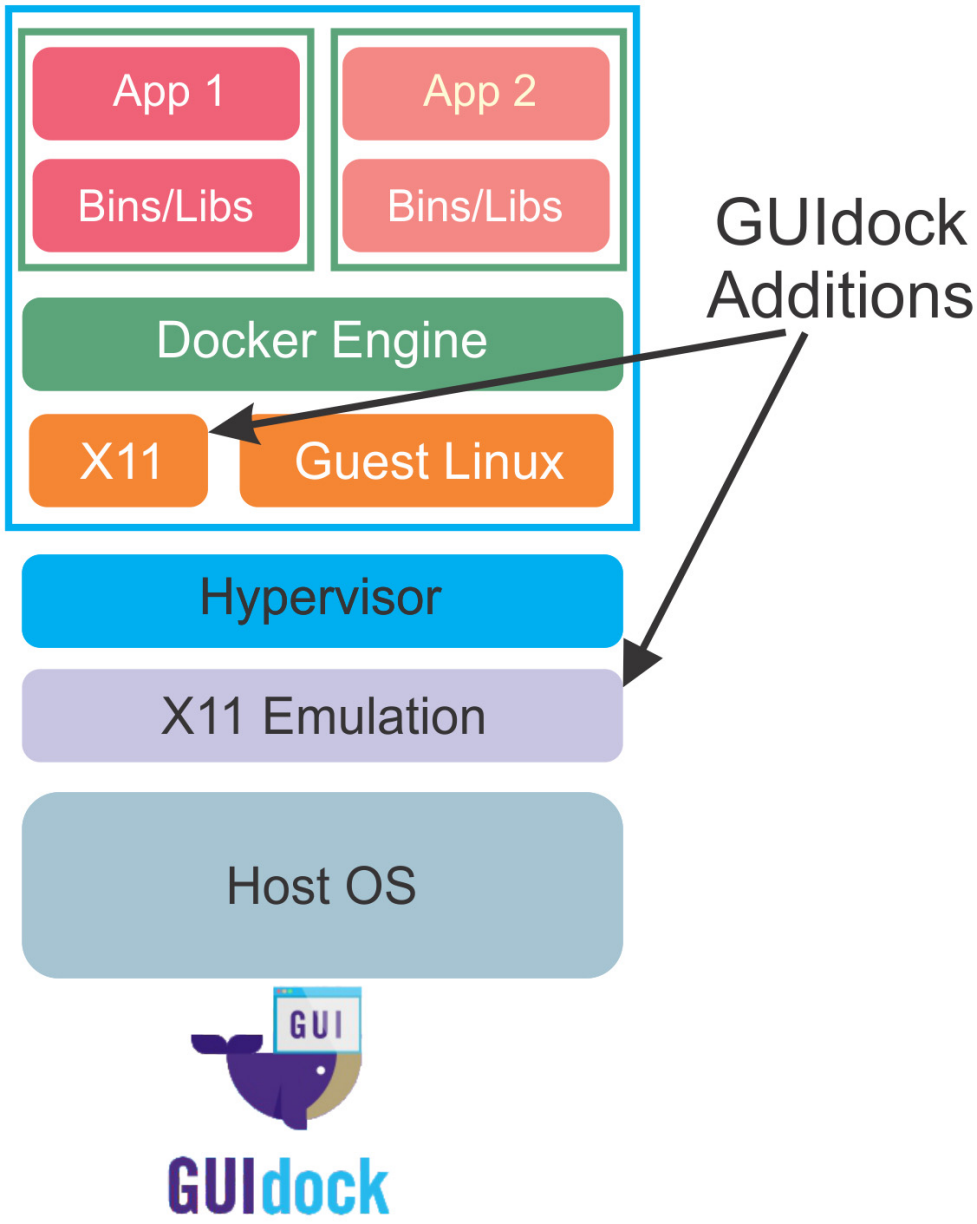
Software components added by GUIdock. Our GUIdock package ensures that the X11 libraries are present in the Linux OS that runs Docker. In the case of a Linux host, this is the host OS. For Windows and Mac OS, this is a guest OS inside a VM. An additional X Windows emulation layer is configured for Windows and Mac OS which allows for GUI commands to be exported from the container software and rendered on the host.

Previous to GUIdock, the workaround when graphics interaction is required has been to distribute WebAPIs that provide a consistent GUI. An example is cyREST [24] which is also available as a Docker package. cyREST provides a RESTful API to Cytoscape and uses a jquery library (cytoscape.js) to render graphics on the different host systems. However, some knowledge of programming is needed to use the API and the result is dependent on the browser and operating system. In contrast, our GUIdock package exports the native Cytoscape GUI into a more consistent X Windows environment. A user simply double-clicks on an icon and the application pops up and is used exactly as it would be when run in its native environment. Fig 2 compares GUIdock to virtual machines and Fig 3 shows the software components added by GUIdock.

## Materials and Methods

A Docker container is essentially a barebones wrapper providing a Linux environment for a set of user applications. Linux distributions use X Windows (X11) as their native GUI. The challenge is to produce the same GUI on hosts that run other operating systems. The solution adopted by GUIdock is to pass the container X Windows commands to a host X Window emulator which renders the GUI. No additional software needs to be included within the container. Everything is done by scripts that are easily modified to install, configure and run any Dockerfile or Docker Image. GUIdock is a truly portable approach that is independent of the contents of the container. Fig 3 summarizes the relationship between various layers, including host operating system, Docker engine, X11 and software applications included in GUIdock.

### Building the CyNetworkBMA GUIdock package

Docker images are reproducible, and it is possible to build a new image from previously established image. Since we have chosen gene network inference as our proof-of-concept example, the Bioconductor Base Image is chosen as the starting point. This image contains R and basic Bioconductor packages. The Dockerfile starts with this image. We then added all the other tools needed to run the CyNetworkBMA app to our Docker image, including R Packages such as Rserve [28], igraph [29], BMA [37], Bioconductor package networkBMA [10], and additional software like java and Cytoscape. We also configured our Docker image to run Rserve in the background before launching Cytoscape.

CyNetworkBMA uses the Cytoscape GUI for user interaction. After the Docker package is created, additional steps are needed to forward the GUI information and render the graphics on the local host. These steps are dependent on the operating system that the local host is running and is automated by GUIdock. For a step-by-step guide on how to deploy a GUIdock package, please refer to the user manual uploaded as S1 Text and in the next section.

### GUIdock: on Linux operating systems

The simplest case is when containers are deployed on Linux systems which use X Windows natively. Since the containers use the same OS as the host, a virtual machine is unnecessary. Instead, the host OS and resources are used and only the necessary supporting libraries and binaries are included in the container. The guest software in the container can also export GUI information and let the host X Windows system render the GUI. These facilities are already provided by Docker. GUIdock uses a simple configurable bash script to automate the installation.

### GUIdock: on Mac OS

Mac OS, like Linux, is a form of UNIX but differs sufficiently that the host OS is not used and a VM is required to encapsulate a guest Linux OS in the container. The creators of Docker have provided Docker machine which uses VirtualBox to create the necessary VM. However, the guest OS cannot directly export GUI commands to the host Mac OS as support for X Windows was dropped in OS X 10.8 (Mountain Lion). Therefore, we use XQuartz [38], an open source project, to provide X11 support on the host. We use socat [39] to bind XQuartz services to an open port and make XQuartz reachable by the Docker container. Socat is a command line based utility that establishes two bidirectional byte streams and transfers data between them. The DISPLAY environment variable in the Docker container is also set to the IP address of the local host running OS X. In this way, the internal X Windows commands are exported to XQuartz which renders the graphics on the host computer. GUIdock provides a set of bash scripts to automate the entire installation, configuration and run process.

### GUIdock: on Microsoft Windows operating systems

The Microsoft Windows operating systems are very different from Linux and use several different proprietary APIs to implement their native GUI. The Windows version of Docker machine, using VirtualBox, provides a VM with Linux for the container. The current Docker toolbox also provides Kitematic, a GUI based manager to deploy containers but not for rendering the GUI from software within containers. We use a lightweight application, MobaXterm [40] for this purpose. Although MobaXterm is proprietary, a full-featured free version is available for download at http://mobaxterm.mobatek.net/download.html. MobaXterm, provides X Windows support and supports ssh (secure shell) tunneling. Ssh is a widely-used UNIX-based command interface and protocol for securely accessing a remote computer. Using MobaXterm, we set up X11 forwarding using ssh to connect the Docker container with a MobaXterm terminal. The GUI commands pass through the ssh tunnel to the MobaXterm X Windows emulator which renders the GUI on the host system. GUIdock provides a double-clickable script to initialize, configure the environment, and run the Docker container.

### Availability and requirements

- Project name: GUIdock
- Project home page: https://github.com/WebDataScience/GUIdock
- Contents available for download: Docker Images, Dockerfiles, installation scripts and execution scripts.
- Operating system(s): Linux, Mac OS X, Microsoft Windows. Specifically, we tested GUIdock on
  - Linux: Fedora, Ubuntu 15.04
  - Mac OS X: 10.9, 10.10
  - Microsoft Windows: 7, 8.1, 10
- Demo video (S1 Video): https://youtu.be/k1WkIx0EENo

## Results

We deployed the CyNetworkBMA GUIdock package and applied it to three different datasets of biological relevance: RNAseq data across human cancer cell lines [41], yeast time series gene expression data [7] and DREAM4 simulated time series data [42]. Note that our demos cover static (non-time series) RNAseq gene expression data in human, time series microarray data in a simple model organism (yeast), and simulated time series data.

**Fig 4:**
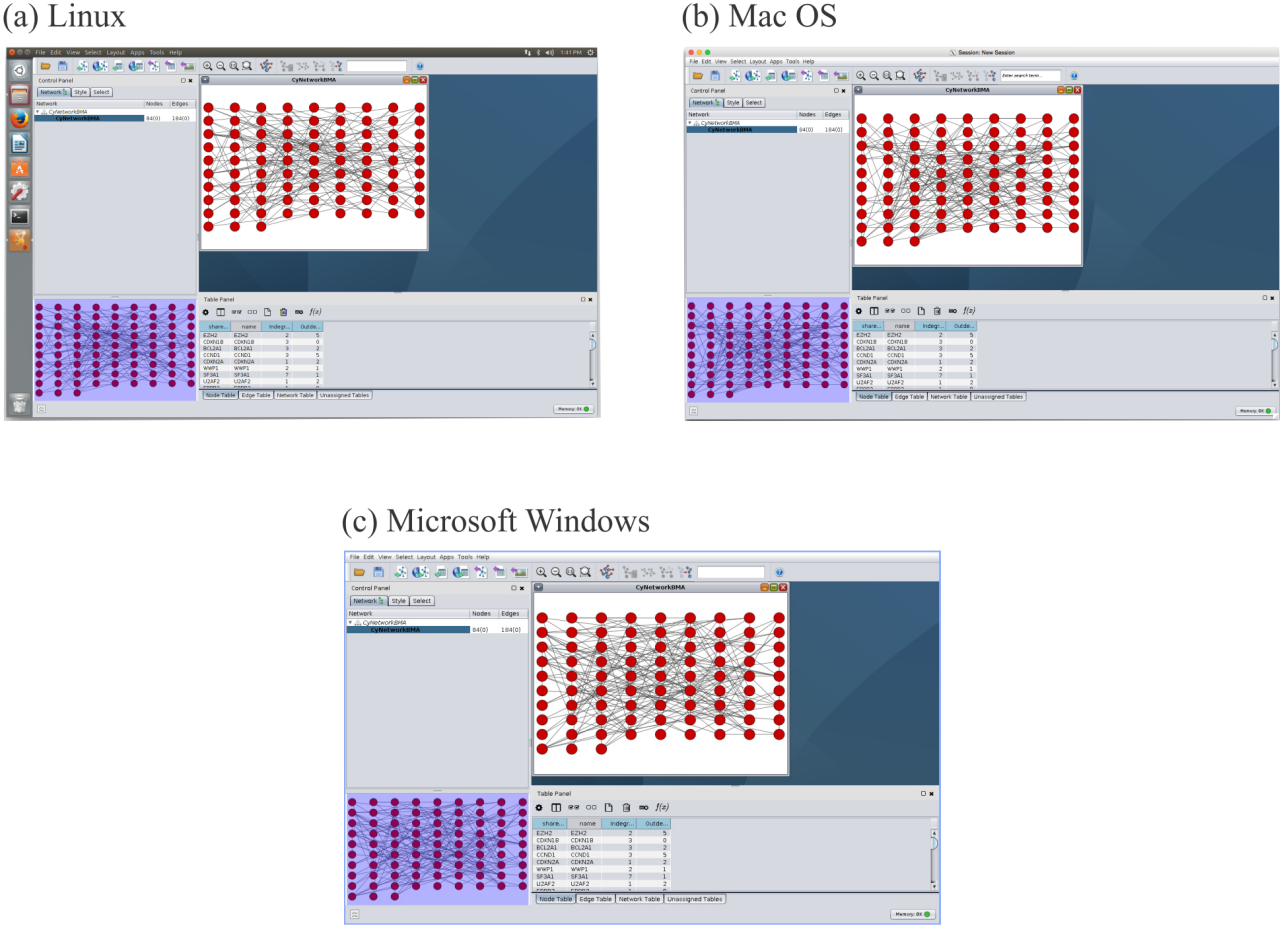
Screen shot of gene networks generated by GUIdock on (a) Linux, (b) Mac OS, (c) Microsoft Windows using the human cancer RNAseq data from Klijn *et al*. Our goal is to demonstrate the reproducibility of analytical results when GUIdock is deployed on computers running different operating systems.

We show that we get identical results after deploying the package on Linux, Mac OS and Windows. We added these test data and results to the GUIdock image. We encourage the readers to download our image and reproduce the results shown in Fig 4.

### Scenario 1: human cancer RNAseq data

Klijn *et al*. generated an extensive RNAseq gene expression data across 675 frequently used human cancer cell lines [41]. We downloaded the variance stabilized version of the normalized RNAseq data produced by the DESeq Bioconductor package [43] from http://research-pub.gene.com/KlijnEtAl2014/. We then extracted a subset of 84 genes that belong to 21 cancer-related pathways that are known to be functionally altered in cancer (see Supplementary Table 12 in Klijn *et al*. [41]). This is a steady-state (non-time series) dataset. We applied the ScanBMA [9] gene network inference algorithm as implemented in the CyNetworkBMA app from within the GUIdock container.

**Fig 5:**
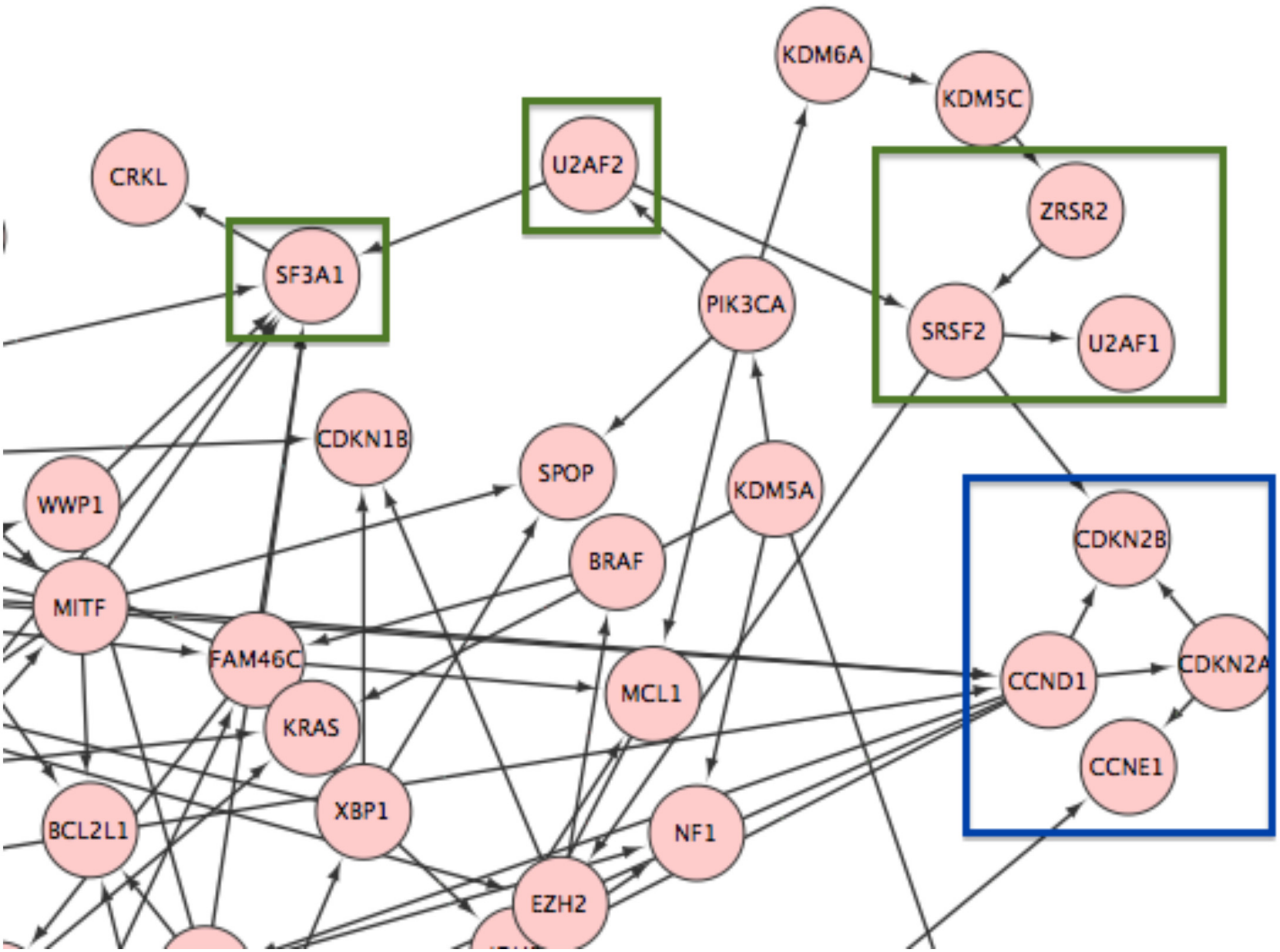
A zoomed-in version of the screen shot of gene network generated by GUIdock using the human cancer RNAseq data from Klijn *et al*. Nodes (CDKN2A, CDKN2B, CCNE1, CCND1) that are part of the cell cycle pathway are highlighted in blue. Nodes (ZRSR2, U2AF1, U2AF2, SRSF2, and SF3A1) that belong to the splicing pathway are highlighted in green.

Fig 4 show the identical results generated by the GUIdock package when installed on a computer running the Linux, Mac OS X and Windows operating systems respectively, after applying CyNetworkBMA to the same Klijn *et al*. cancer cell line RNAseq data. We demonstrate the reproducibility of analytical results when GUIdock is deployed on local hosts running different operating systems. Fig 5 shows a zoomed in sub-graph of the same network. From Fig 5, we observe inferred edges among nodes (CDKN2A, CDKN2B, CCNE1, CCND1) that are part of the cell cycle pathway as indicated in Supplementary Table 12 in Klijn *et al*. [41]. Similarly, we also observe inferred edges among nodes (ZRSR2, U2AF1, U2AF2, SRSF2, and SF3A1) that belong to the splicing pathway.

### Scenario 2: Yeast time series microarray data

We also deployed the GUIdock package and applied CyNetworkBMA to a subset of the yeast time series data [7]. Yeung *et al*. profiled the response of 97 yeast segregants over six time points subjected to rapamycin perturbation using microarrays. We extracted a 100-gene subset and applied CyNetworkBMA using all default settings. Similar to the previous subsection, we deployed GUIdock on both Windows and mac. See S1 Text for screenshots.

### Scenario 3: DREAM 4 simulated data

The DREAM4 simulated time series data consist of 100 genes over 21 time points [42]. We deployed the GUIdock package on Linux, Mac and Windows. See S1 Text for screenshots.

## Discussion

In academic bioinformatics research, data is usually collected and analyzed using a suite of multiple software tools, implemented by different developers using different languages. Most workflows are not completely command-line based and have a GUI component. Gathering and compiling the software components is not a trivial task and is *not* always sufficient to reproduce identical results as reported in the scientific literature. GUIdock uses Docker containers to replicate and distribute the original workflow.

We used gene network inference as a proof-of-concept example. Distribution of the CyNetworkBMA app was non-trivial because the user is required to install and set up dependencies such as Rserve, even though the app itself was written in portable java and Cytoscape runs natively on different platforms. In our figures and demo videos (S1 and S2 Videos), we show that by using GUIdock, the user only has to run a provided script to replicate the original environment and reproduce the results.

Bioinformatics often have to deal with big data that are stored in the cloud. The modular Docker container repository paradigm is particularly well suited for building cloud based applications. In the future, we plan to extend GUIdock to work with Docker containers deployed in the cloud and have done some preliminary work on configuring Microsoft Azure instances and with multiple containers. We also plan to study the performance impact of adding the X11 layer to GUIdock in various operating systems.

The primary purpose of this manuscript is to provide a proof of concept for using the GUIdock methodology to reproduce bioinformatics results. The choice of software was primarily driven by robustness and the ease of installation and use by the end user. Another obvious application for container methods is the construction and deployment of custom pipelines from individual modules rather than from downloading an entire application suite. In this case, performance becomes a consideration and we may need to reconsider some design choices such the X-emulator used and the use of a ssh tunnel to transfer X Windows information.

To summarize, we have developed GUIdock, a new container based workflow to deploy GUI based pipelines. We have demonstrated the effectiveness of GUIdock by using it to deploy our Cytoscape based CyNetworkBMA app on Linux, Mac OS and Windows host systems. We have provided scripts to automate the installation and deployment of GUIdock packages. We anticipate that GUIdock will be an important step in solving the problem of testing and reproducing scientific results that come from ever-increasingly complicated multi-component software.

## Supporting Information

S1 Text User manual for GUIdock. In this user manual, we describe the use of our scripts to install and run GUIdock on Linux, Mac OS and Windows. In addition, we provide a step-by-step guide to document each step in the installation and deployment process. We also included additional screen shots for the demos described in the Results section.
(PDF)

S1 Video Demonstration of GUIdock on Linux, Mac OS and Windows. In this video, we ran GUIdock on the same sample dataset across Linux, Mac OS and Windows. We demonstrate that identical gene networks were derived in each operating system. We chose a 9-gene subset of the human cancer RNAseq data from Klijn *et al*. [41]. Among these 9 genes, (CDKN2A, CDKN2B, CCNE1, CCND1) belong to the cell cycle pathway and (ZRSR2, U2AF1, U2AF2, SRSF2, SF3A1) belong to the splicing pathway as indicated in Supplementary Table 12 in Klijn *et al*. [41]. This video is also available on YouTube at https://www.youtube.com/watch?v=k1WkIx0EENo.
(MOV)

S2 Video Installation of GUIdock on Linux. In this video, we showed the steps involved in installing GUIdock on computers running Linux. This video (no voice) is available on YouTube at https://www.youtube.com/watch?v=HOtI1Eg2J1Q. A version with audio is available on YouTube at https://www.youtube.com/watch?v=-CrfhxNuMgc&index=2&list=PLczI6k_oOIdbZQTMTMRcD9QmfWCXAuLsd.
(MOV)

S3 Video Installation of GUIdock on Mac OS. In this video, we showed the steps involved in installing GUIdock on computers running Mac OS. This video is also available on YouTube at https://www.youtube.com/watch?v=4Qg0fCDOxhY.
(MOV)

S4 Video Installation of GUIdock on Windows. In this video, we showed the steps involved in installing GUIdock on computers running Windows. This video is also available on YouTube at https://www.youtube.com/watch?v=cA7HVCB064I.
(MOV)
