# Supplementary material for: GUIdock: Using Docker Containers with a Common Graphics User Interface to Address the Reproducibility of Research
Source: PLoS One. 2016 Apr 5;11(4):e0152686. doi: 10.1371/journal.pone.0152686 (PMC4821530; doi:10.1371/journal.pone.0152686)

GUIdock

USER MANUAL

Latest version available at https://github.com/WebDataScience/GUIdock

---

GUIdock is a Docker Package containing the entire computational environment to run applications with a graphical user interface. As a proof of concept, we focus on supporting Cytoscape, a Java-based standalone program with a graphical user interface for the visualization and analyses of gene networks in biology. In addition to Cytoscape, our container includes R, Rserve, Bioconductor packages igraph, networkBMA and a Cytoscape app called CyNetworkBMA. GUIdock contains the entire pipeline and all the tools (including Cytoscape that connects to preloaded RServe, a Bioconductor package called "networkBMA" and all its dependencies) to generate gene regulatory networks.

This manual demonstrates the installation and instructions to run GUIdock.

Docker Image : https://hub.docker.com/r/kristiyanto/guidock/
Dockerfile : https://github.com/WebDataScience/GUIdock

Authors

Ling-Hong Hung, Daniel Kristiyanto, Sung Bong Lee, Ka Yee Yeung

Institute of Technology
University of Washington
Tacoma, WA, 98402, USA

Table of Contents

- Quick Start
  - Linux
    - Running GUIdock (SCRIPT)
  - OS X
    - Installation (SCRIPT)
    - Running GUIdock (SCRIPT)
  - WINDOWS
    - Installation (SCRIPT and MANUAL)
      - Docker ToolBox
      - MobaXterm
    - Running GUIdock (SCRIPT)
- Manual
  - OS X
    - Installation (MANUAL)
      - Docker Toolbox
      - Home Brew
      - XQuartz
      - Socat
    - Running GUIdock (MANUAL)
      - Preparation
      - Running GUIdock
  - WINDOWS
    - Installation (MANUAL)
    - Running GUIdock (MANUAL)
      - Preparation
      - Running GUIdock
- Cytoscape Demo
  - Open Demo Session
  - Inferring Gene Networks
  - Transferring files between a container and the local filesystem
    - Transferring files (Linux)
    - Transferring files (OS X)
    - Transferring files (Windows)
      - Transferring a file from the local filesystem to running container
      - Transferring a file from running container to the local filesystem

Quick Start

Linux

Running GUIdock (SCRIPT)

A bash script to run and install GUIdock is available in:
https://github.com/kristiyanto/GUIdock/tree/master/Install%20and%20Run%20/LINUX

Download a file from the link above and run the script with following command to start GUIdock.

```
#Locate to script file
#Use following command to start GUIdock

sh runGUIdockLinux.sh
```

Alternatively, a tarball file is also available.

The script and tarball will attempt to start Docker Engine or install it if it is not installed. Once started, it pulls the Docker Image and run it.

During the run, a folder .guidock/GUIdock-SHARED is created under user home directory. ~/.guidock/GUIdock-Shared in the Linux system is linked with /root/GUIdock-SHARED within the container.

OS X

Installation (SCRIPT)

As shortcut, a bash script to run installation commands below is available in:
https://github.com/WebDataScience/GUIdock/tree/master/Install%20and%20Run%20/MAC

Once downloaded, unzip a file and run the script with following command to install all the required softwares.

```
# Install from script

sh install-mac.sh
```

The script, however does not install Docker Toolbox. Docker Toolbox will have to be downloaded from their website and installed separately. Docker Toolbox can be downloaded from: https://www.docker.com/toolbox.

Running GUIdock (SCRIPT)

A script to run GUIdock is available in:
https://github.com/WebDataScience/GUIdock/tree/master/Install%20and%20Run%20/MAC

Download a file from the link above and unzip a file.

To run GUIdock, run the script using the following command:

```
# Download and locate to installation file.
# Use following command to start GUIdock

sh mac-start.sh
```

WINDOWS

Installation (SCRIPT and MANUAL)

1) Docker ToolBox

Install Docker ToolBox. Docker ToolBox can be downloaded from:
https://www.docker.com/toolbox

Click the link above and choose "Download (Windows)".

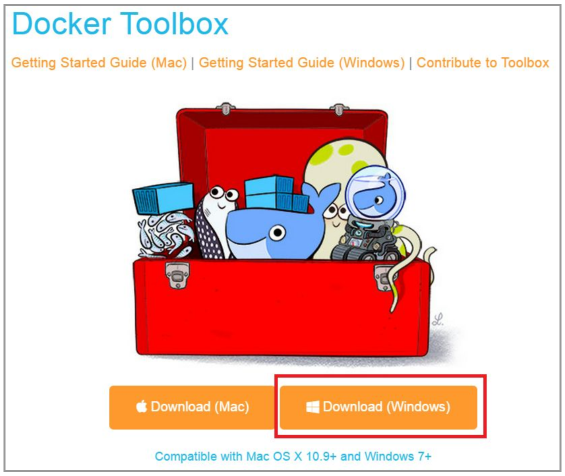

Go to folder where the installation file is saved and run the installation file.

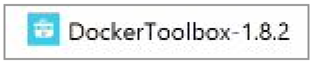

Click "Next" until you see "Selection Components" window. This will install Docker client, Docker machine, VirtualBox, Kitematic and Git for Windows.

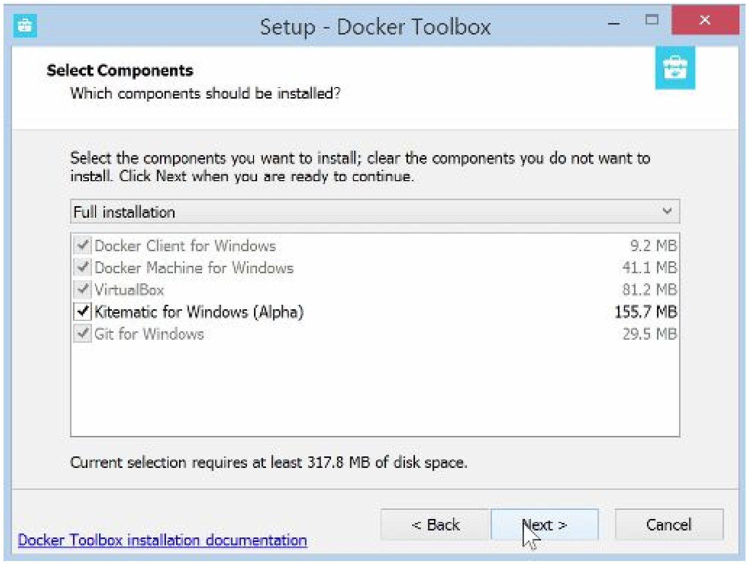

Click “Next” until you see “Ready to Install” window and click “Install”.

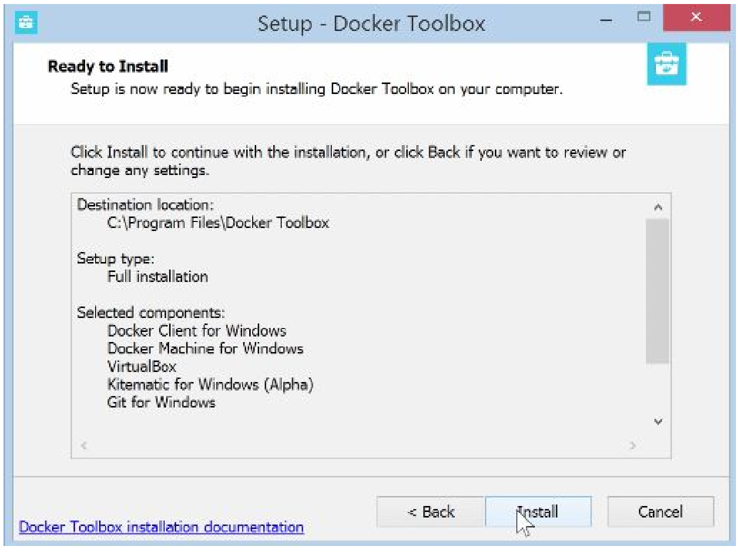

Click "Finish" to complete the installation.

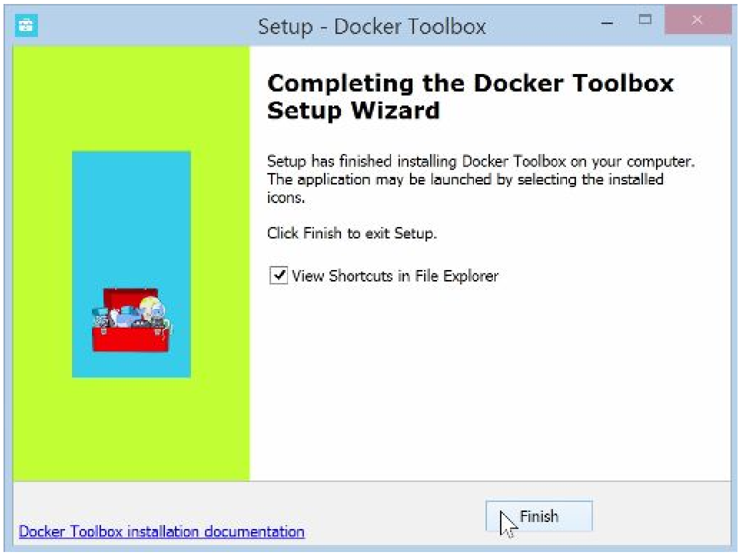

To run the Docker Engine, double click on "Kitematic (Alpha)" shortcut.

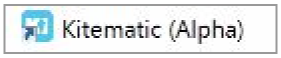

On the first run, it will create the Docker VM and all the required environments. If you have a Docker account, enter your username and password. If you don't have an account, click "Skip for now".

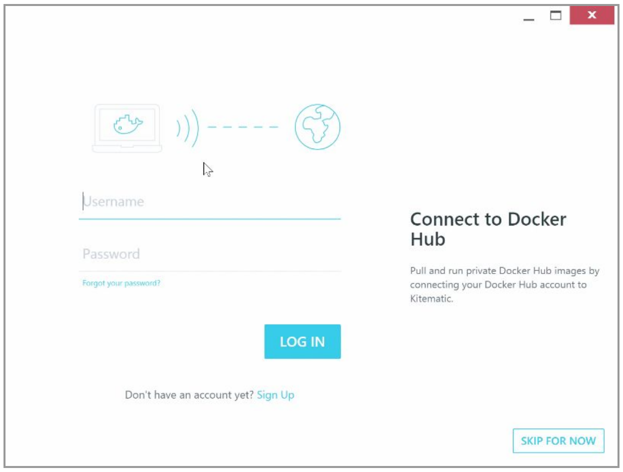

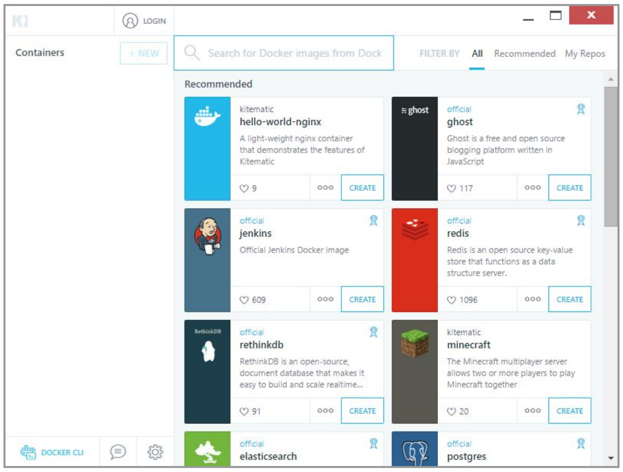

2) MobaXterm

To have GUI delivered in Windows, install MobaXterm to set up X11 forwarding. MobaXterm is an open source X11 emulator for Windows which allows remote GUI access to the application. MobaXterm can be downloaded from:
http://mobaxterm.mobatek.net/download-home-edition.html
Click the link above and choose version you want to download. We chose “MobaXterm Home Edition (Installer edition)” in this tutorial.

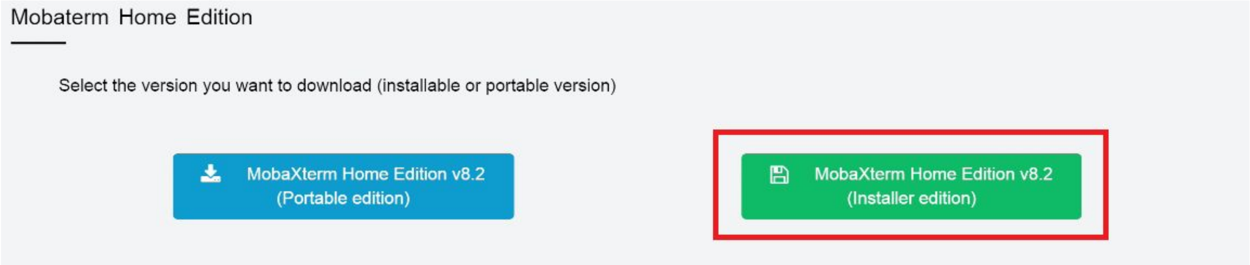

Go to folder where the installation file is saved and run the installation file.

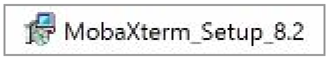

Click “Next” until you see “Ready to install MobaXterm” and click “Install” to install MobaXterm.

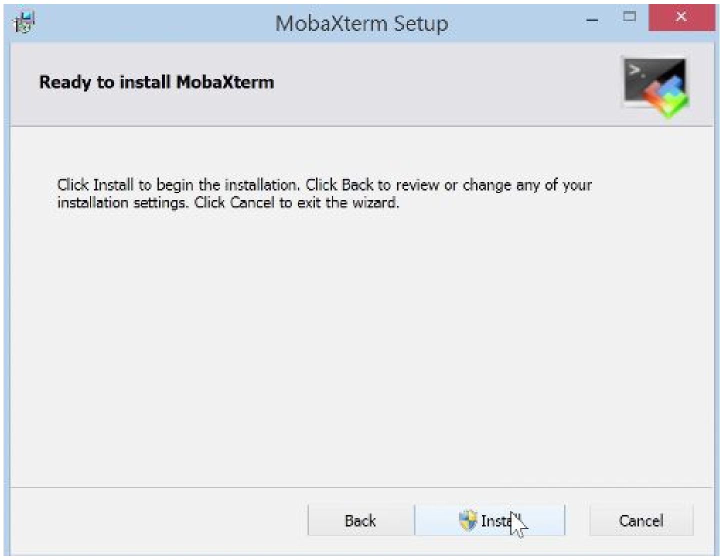

Running GUIdock (SCRIPT)

A shell script to run GUIdock is avilable in:
https://github.com/WebDataScience/GUIdock/tree/master/Install%20and%20Run%20/WINDOWS

Download a file from the link above and unzip a file. Double click on "RunGUIdock.sh" shell script file to run GUIdock. The script, however does not install Docker Toolbox and MobaXterm. Docker Toolbox and MobaXterm will have to be downloaded from their websites and installed separately.

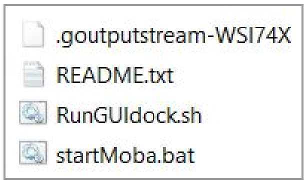

Manual

OS X

Installation (MANUAL)

Alternatively, required packages can also be installed independently by running following instructions:

1) **Docker Toolbox**

Docker Toolbox contains Docker Virtual Machine and Kitematic as a Docker Client for Macintosh and Windows User. Docker Toolbox can be downloaded from: https://www.docker.com/toolbox

2) **Home Brew**

Using only Docker Toolbox gives all that Docker Engine promises. However, to have GUI delivered in Mac from Docker containers, more software are needed. To make installation easier, software can be installed using home brew (http://brew.sh/).

To install Home Brew, enter this command in OS X terminal window:

```
# Install Home Brew

ruby -e "$(curl -fsSL
https://raw.githubusercontent.com/Homebrew/install/master/insta
ll)"
```

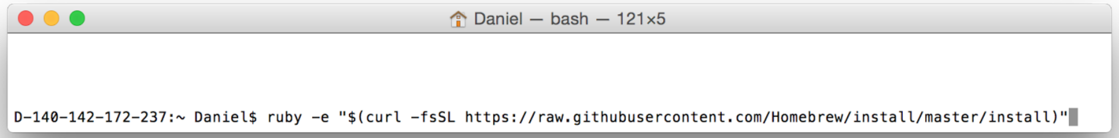

3) **XQuartz**

XQuartz is an open source program to enable X Windows on OS X. With Home Brew installed, XQuartz can be installed entering this command in OS X terminal window:

```
# With home brew installed, install xquartz
brew install xquartz
```

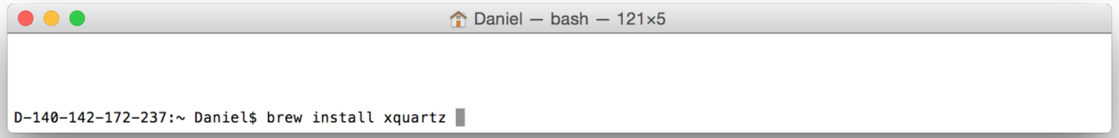

4) Socat

XQuartz is an open source program to enable X Windows on OS X. With Home Brew installed, enter the following command in terminal window:

```
# With home brew installed, install socat
brew install socat
```

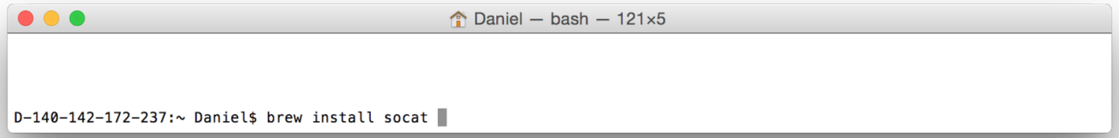

Running GUIdock (MANUAL)

Alternatively, GUIdock can also be launched manually by following instructions below:

1) Preparation

Docker engine runs in a virtual machine in OS X. To enable Display to be delivered to Mac OS, use socat to open incoming X11 Graphic interaction from Docker Machine and Xquartz to display the graphics.

```
# Run to socat bind
socat TCP-LISTEN:6000,reuseaddr,fork UNIX-CLIENT:\"$DISPLAY\"
```

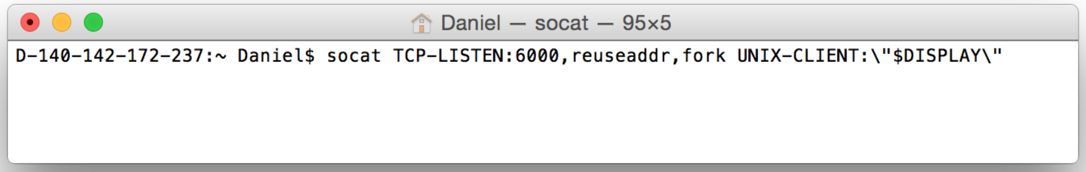


```
# Open xquartz to accept incoming graphic
open -a xquartz
```

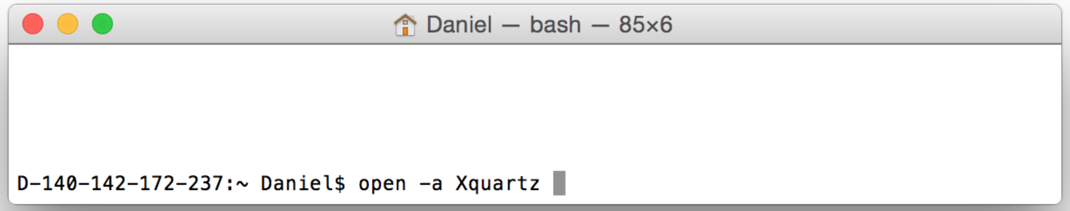

It may also a good idea to check if the firewall is blocking the incoming connection from docker container.

2) Running GUIdock

Once Mac OS is ready to receive graphic streams from Docker container, ensure that Docker Engine (Kitematic) is up and running. Click on the Docker CLI in Kitematic to launch a new terminal

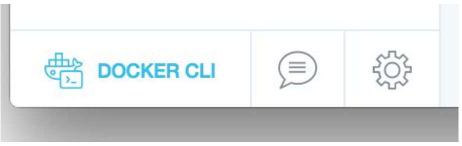

Within Docker CLI terminal windows, run GUIdock by running following commands:

```
# Pull latest image of GUIdock from repository.
docker pull kristiyanto/guidock
```

```
#  Find out the Mac's IP address
ifconfig | grep "inet"
```

The MAC-IP-ADDRESS number you want to identify is immediately after the "inet" field.

```
# Run the GUIdock. Once loaded, a Cytoscape GUI Window pops up.
# Replace MAC-IP-ADDRESS with the IP number you identified from ifconfig

docker run -ti -e DISPLAY=MAC-IP-ADDRESS:0 kristiyanto/guidock
```

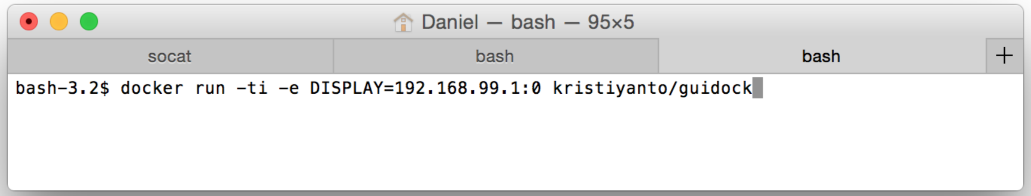

-ti : To enable docker container in interactive mode.
-e : To set up the DISPLAY environment variable into the docker container so that the GUI can be sent to Mac OS machine.
IP address : MAC OS private address (VirtualBox's interface), this address should be reachable by Docker Machine.
Port Number : Port number of X11 is 0 (zero).

Next: Cytoscape Demo

WINDOWS

Installation (MANUAL)

Please refer to the Quick Start installation page on how to install Docker ToolBox and MobaXterm.
Installation (SCRIPT and MANUAL)

Running GUIdock (MANUAL)

1) Preparation

Firewall
Make sure Firewall allows Docker Machine to access MobaXterm X11 service. This can be done either by disabling the firewall for Private Networks, or by manually list MobaXterm in Firewall as follow:

Control Panel -> System and Security -> Windows Firewall -> Allow an app or feature through Windows Firewall; check boxes for MobaXterm

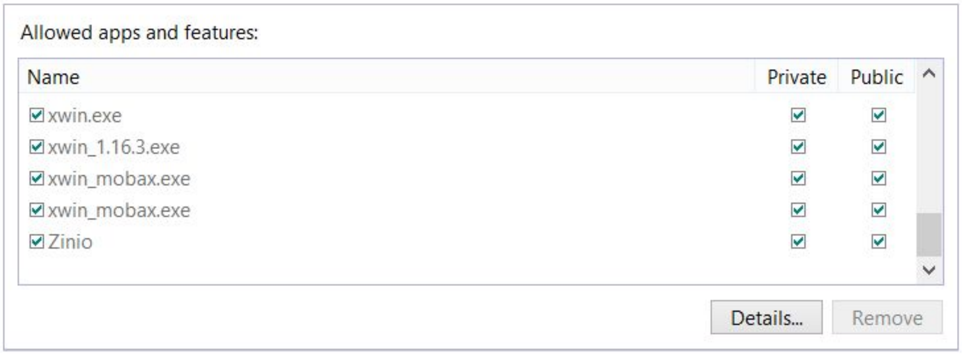

IP Addresses
Open “Docker QuickStart Terminal”.

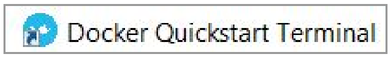

To obtain IP Addresses, type the following command in “Docker QuickStart Terminal”:

```
docker-machine inspect default
```

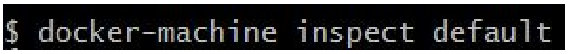

Locate to the IP Address of Docker Machine.

```
"IPAddress": "192.168.99.100",
```

Locate to IP Address of Docker Client.

```
"HostOnlyCIDR": "192.168.99.1/24"
```

2) Running GUIdock

Open MobaXterm, click "Start local terminal".

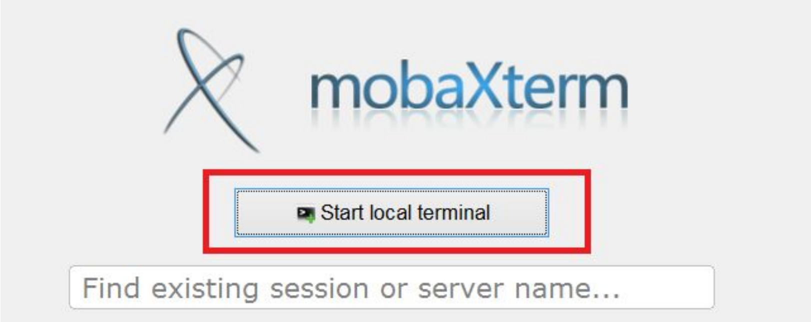

Type the following command to use SSH to connect Docker host in terminal.

```
# 1. SSH with X11 forwarding to Docker host.
ssh -X docker@DockerMachineIPAdress
```

```
ssh -X docker@192.168.99.100
```

Docker default username is "docker" and password is "tcuser". Enter "tcuser" when a password is asked. Password can be saved after the first login.

```
#2. Login with password "tcuser"
tcuser
```

```
docker@192.168.99.100's password:
```

Pull the latest GUIdock image from the repository by typing the following command in terminal:

```
# 3. Pull latest image of GUIdock from repository.
docker pull kristiyanto/guidock
```

```
docker@default:~$ docker pull kristiyanto/guidock
```

Run GUIdock image by typing the following command in terminal

```
#4. Run the GUIdock. Once loaded, a Cytoscape GUI Window pops up.

docker run -ti -e DISPLAY=WindowsIPAddress:0
kristiyanto/guidock
```

```
docker@default:~$ docker run -ti -e DISPLAY=192.168.99.1:0 kristiyanto/guidock
```

-ti : To enable docker container in interactive mode.
-e : To set up the DISPLAY environment variable into the docker container so that the GUI can be sent to Windows machine.
IP address : Windows private address (VirtualBox's interface), see Preparation Section to check IP Address. This address should be reachable by Docker Machine.
Port Number : Port number of X11 is 0 (zero).

Next: [Cytoscape Demo]

Cytoscape Demo

Demo files and saved-sessions are included in the GUIdock container, located in /root/DEMO folder.
The files and the demo including:

1. Dream4.cys : A 210 by 101 table in which the first column identifies time and the remaining columns are the expression levels for 100 genes in a simulated network after an application (at t=0) and removal (at t=500) of a perturbation (21 time points in each time series). Based on the time series file for the first network in the 100-gene in silico network sub-challenge from DREAM 4, Challenge 2 (Marbach *et al.* 2009, Marbach *et al*. 2010, Prill *et al.* 2010). A key column was added to the original file to facilitate import into Cytoscape.
2. RNAseq.cys : 84-gene subset of the RNAseq Cancer cell line data. Klijn *et al.* generated an extensive RNAseq gene expression data across 675 frequently used human cancer cell lines. We downloaded the the variance stabilized version of the normalized RNAseq data produced by the DESeq Bioconductor package from http://research-pub.gene.com/KlijnEtAl2014/. We then extracted a subset of 84 genes that belong to 21 cancer-related pathways that are known to be functionally altered in cancer (see Supplementary Table 12 in Klijn *et al*. Note that this is a steady-state (non-time series) dataset.
3. Yeast.cys : A 582 by 102 table in which the first two columns identify the replicate and time (in minutes) after drug perturbation, and the remaining 100 columns are the expression measurements for a subset of 100 genes from the yeast-rapamycin experiment described in Yeung et al. (2011). There are 582/6 = 97 replicates (the 95 segregants plus two parental strains of the segregants), each with measurements at 6 time points. The complete time series data is available from Array Express (Parkinson et al. 2007) with accession number E-MTAB-412. When using this file, please remember to adjust source format in the inference dialog. Also, exclude replicate and time columns from input to the algorithm.

Open Demo Session

Once Cytoscape application is started, click "File" and "Open" to open one of the session files from "/root/DEMO" folder.

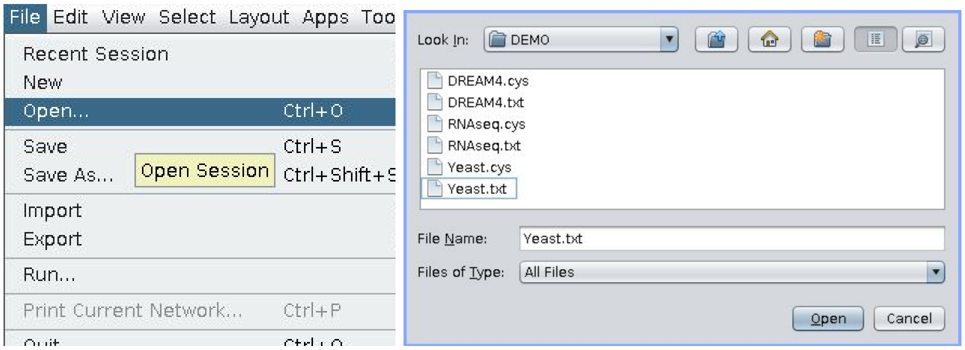

Select any of the .cys file to open saved cytoscape session. The following image is gene networks of RNAseq.cys file.

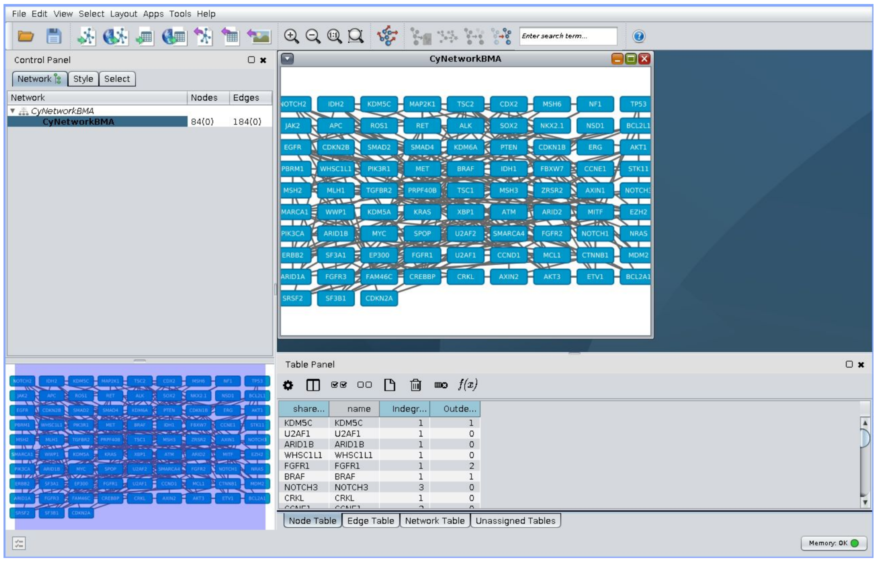

Inferring Gene Networks

Demo files can also be imported into Cytoscape by using the Import menu. On Cytoscape, open test data by going to File -> Import -> Table -> File

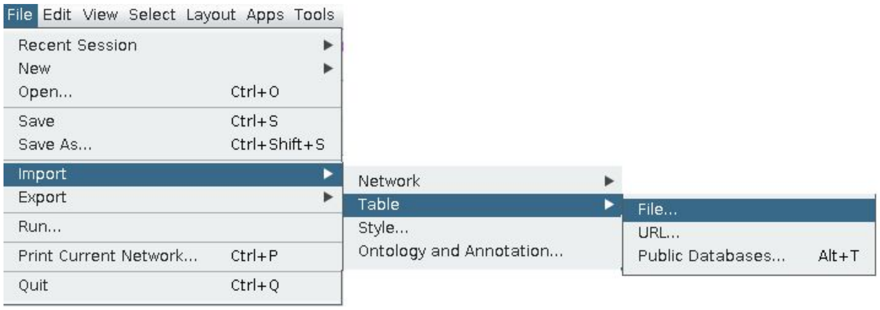

And select any of the .txt files to load demo files into Cytoscape. The files are located in "/root/DEMO"

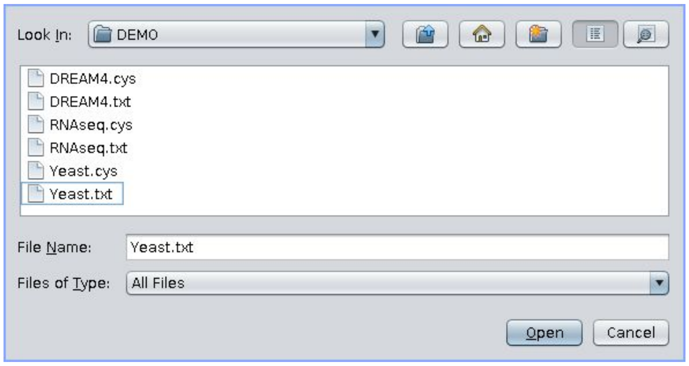

In the following dialog window, choose the option to import data into an unassigned table and enter new table name.

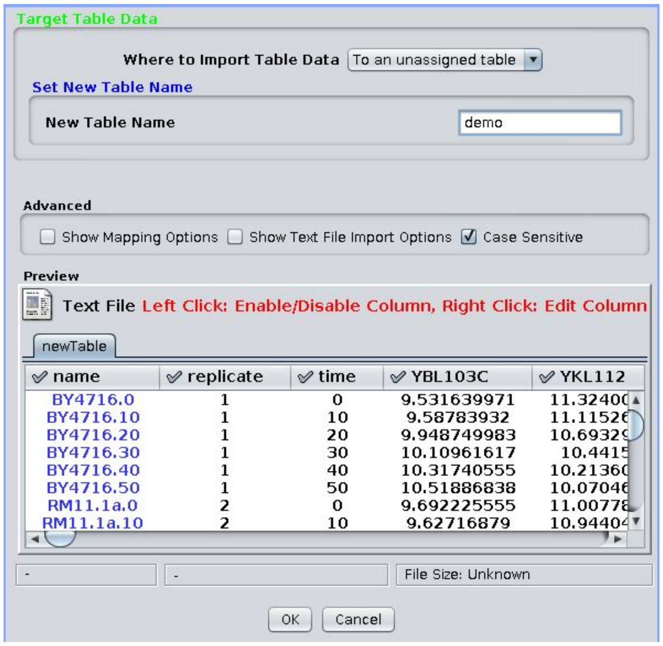

Run the network inference algorithm by going to Apps -> CyNetworkBMA -> Inter Network

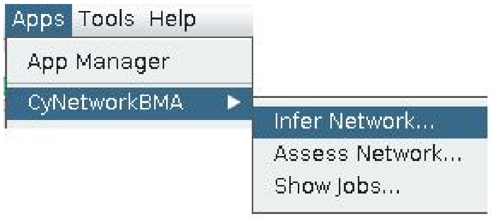

In the following dialog window, specify the columns to include and source format.

Data file	Columns to include	Source format
Dream4.txt	Select all columns except "Time"	● Genes as columns<br>● Data Type: Time series<br>● Number of time points: 21
RNAseq.txt	Select all	● Genes as rows<br>● Data Type: Steady state
Yeast.txt	Select all columns except "replicate" and "time"	● Genes as columns<br>● Data Type: Time series<br>● Number of time points: 6

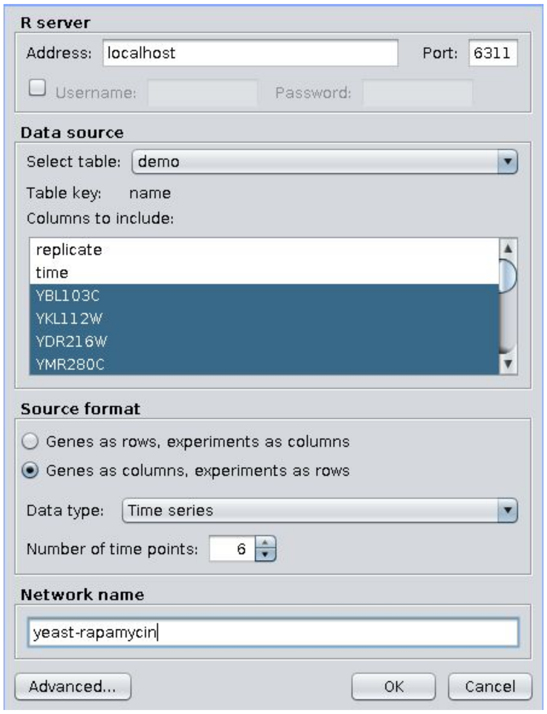

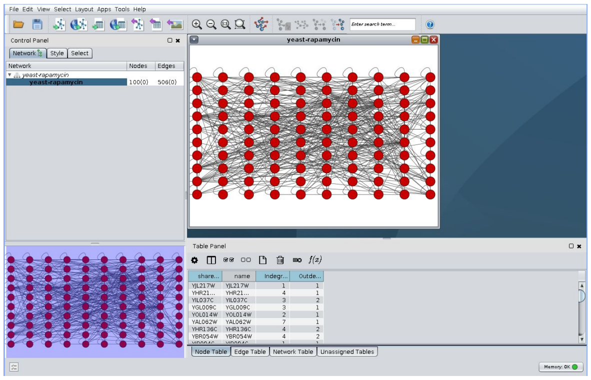

Transferring files between a container and the local filesystem

Transferring files (Linux)

To transfer files in Linux, run the script using the following command to start GUIdock.

```
#Locate to script file
#Use following command to start GUIdock

sh runGUIdockLinux.sh
```

The script creates a shared folder ".guidock/GUIdock-SHARED" under user home directory. ~/.guidock/GUIdock-Shared in the Linux system is linked with /root/GUIdock-SHARED within the container.

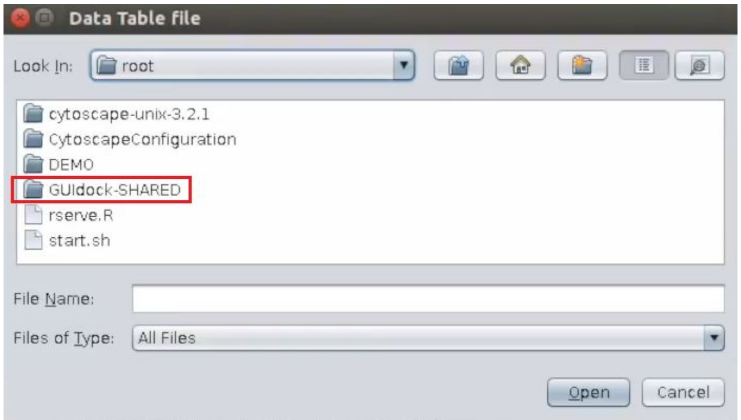

To open shared folder in Linux system, type the following commands in terminal:

```
#Locate to .guidock directory
cd .guidock/
```

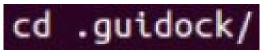


```
#Open shared folder
xdg-open .
```

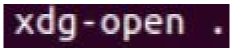

Double click on the shared folder "GUIdock-SHARED".

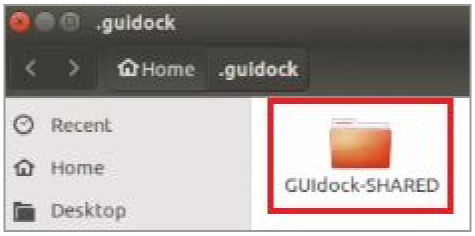

In shared folder, copy and paste a file or folder you want to share. The folder is added to the "/root/GUIdock-SHARED" directory in the container.

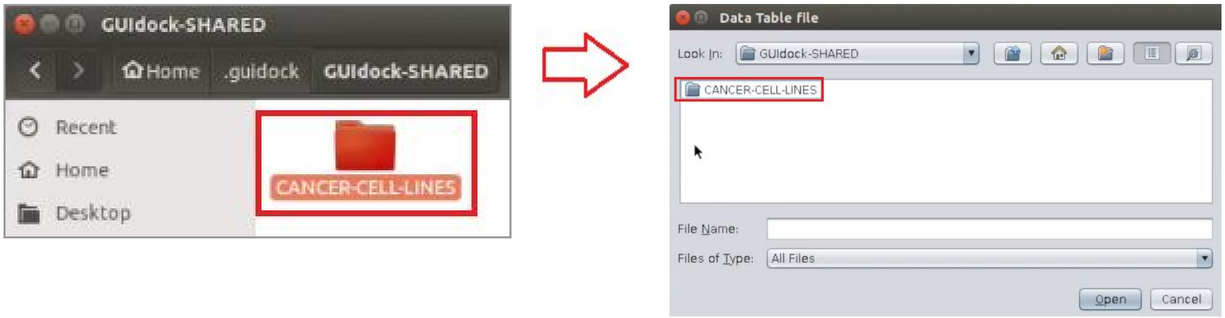

To share files or folders between a container and Linux system, add them in "GUIdock-SHARED" folder.

Transferring files (OS X)

To transfer files between a container and the local filesystem in OS X, run the script using the following command to start GUIdock.

```
# Download and locate to installation file.
# Use following command to start GUIdock

sh mac-start.sh
```

Open terminal and follow the steps for "**Transferring a file from the local machine to running container"** and **"Transferring a file from running container to the local filesystem**
" below.

Transferring files (Windows)

To transfer files between a container and the local filesystem in Windows, double click on the shell script file to start GUIdock.

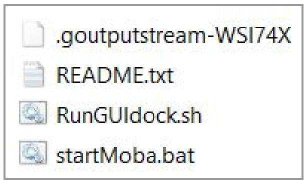

Open "Docker QuickStart Terminal" and follow the steps for "**Transferring a file from the local machine to running container"** and **"Transferring a file from running container to the local filesystem**" below.

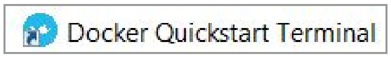

Transferring a file from the local filesystem to running container

Check running container by typing the following command in terminal :

```
#List running containers

docker ps
```

```
$ docker ps
CONTAINER ID        IMAGE                                    COMMAND                  CREATED
        STATUS              PORTS                    NAMES
da229e046dc8        kristiyanto/guidock    "/bin/sh -c 'sh /root"   35 minutes ag
o       Up 35 minutes       8787/tcp                 gloomy_bardeen
```

Transfer a file from the local filesystem to running container by typing the following command in terminal:

```
#Transfer file from the local filesystem to a container.

docker cp localPath containerID:path
```

```
$ docker cp Desktop/CANCER-CELL-LINES/CancerCellLine.txt da229e046dc8:/root/DEMO
```

Data file is added to "/root/DEMO" directory.

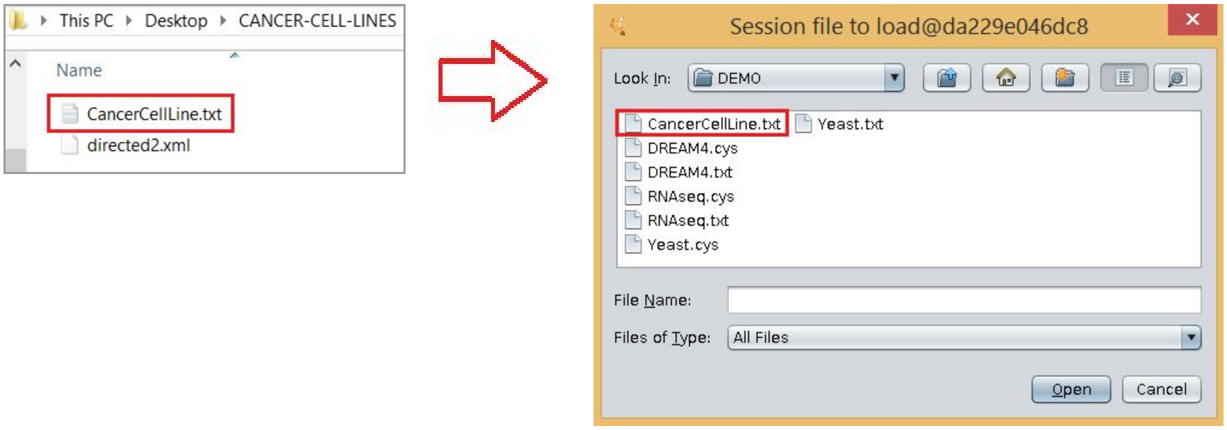

Transferring a file from running container to the local filesystem

To transfer a file from running container to the local filesystem, type the following command in terminal:

```
#Transfer file from a container to the local filesystem.
docker cp containerID:path localPath
```

```
$ docker cp da229e046dc8:/root/DEMO/Test.cys Desktop/CANCER-CELL-LINES/
```

Data file is saved in "Desktop/CANCER-CELL-LINES" directory from running container.

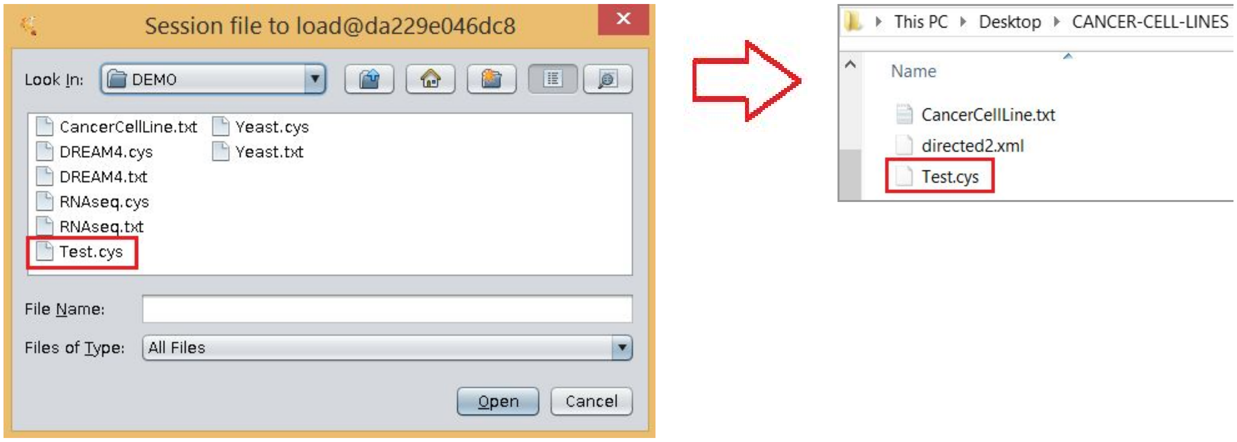

Supplement: S1 Text — In this user manual, we describe the use of our scripts to install and run GUIdock on Linux, Mac OS and Windows. In addition, we provide a step-by-step guide to document each step in the installation and deployment process. We also included additional screen shots for the demos described in the Results section. (PDF) [file pone.0152686.s001.pdf]
